# The accuracy of respiratory rate assessment by doctors in a London teaching hospital: a cross-sectional study

**DOI:** 10.1007/s10877-014-9621-3

**Published:** 2014-10-02

**Authors:** Keir E. J. Philip, Emma Pack, Valentina Cambiano, Hannah Rollmann, Simon Weil, James O’Beirne

**Affiliations:** 1 Royal Free London NHS Foundation Trust, London, UK; 2 University College London, London, UK; 3 85 Effingham Road, St. Andrews, Bristol, BS6 5AY UK

**Keywords:** Respiratory rate, Vital signs, Observations, Assessment

## Abstract

Respiratory rate (RR) is one of the most sensitive markers of a patient condition and a core aspect of multiple clinical assessment tools. Doctors use a number of methods to assess RR, including formal measurement, and ‘spot’ assessments, although this is not recommended. This study aimed to assess the accuracy of the methods of RR measurement being used by doctors. A cross-sectional study assessing the accuracy (range, bias, and imprecision) of doctors’ ‘spot’ and ‘formal’ respiratory rate assessments, using videos of mock patients. 54 doctors in a London teaching hospital participated. Both methods showed high levels of inaccuracy, though formal methods were more accurate than ‘spot’ assessments. 52 and 19 % of doctors did not identify the respiratory rates shown as abnormal, using ‘spot’ and formal assessment methods respectively. We observed a trend towards decreasing accuracy of ‘spot’ assessments with increasing clinical experience (*p* = 0.0490). Current methods of RR assessment by doctors are inaccurate. This may be significantly delaying appropriate clinical care, or even misguiding treatment.

**Electronic supplementary material:**

The online version of this article (doi:10.1007/s10877-014-9621-3) contains supplementary material, which is available to authorized users.

## Introduction

Respiratory rate (RR) is a vital component of clinical assessment and monitoring. The National Institute for Clinical Excellence (NICE) state that RR is the most sensitive marker of a deteriorating patient and the first observation to indicate a problem [1]. Research has shown that abnormalities in RR predict serious adverse events including cardiac arrest and ITU admission [2–5]. Poor clinical monitoring has been highlighted as a principal contributor to avoidable mortality in English hospitals, implicated in 31 % of preventable deaths [6].

Due to its clinical importance RR is an integral component of multiple clinical assessment systems such as Early Warning Systems (EWS) [4, 5]; the Systemic Inflammatory Response Syndrome (SIRS) [7]; and the assessment of acute asthma [8]. RR measurement has multiple clinical applications including: to gain a baseline for comparison; monitor fluctuations; recognise acute changes in a patient’s condition; sign of deterioration; effectiveness of response to treatment; recognition of need for escalation; post-operative comparisons to base line; and the recognition of transfusion reactions [9]. Importantly, assessments are made by multiple healthcare workers during a patients hospital stay, increasing the importance of standardised, accurate methods of assessment. The various uses of RR recordings facilitate appropriate responses to a patient’s condition. Subsequently assessments must be accurate and inaccuracies may delay responses or even misguide clinical care.

Research has shown emergency department triage nurses’ assessments have low sensitivity in detecting bradypnoea and tachypnoea, and show poor agreement with criterion standard measurements by researchers [10]. Furthermore, a recent study showed clinical staff have low levels of confidence in the accuracy of RR measurements in observation charts, believing rates are estimated, or even fabricated, and not formally assessed using recommended methods. Staff also reported using ‘spot’ assessments of RR, in which they estimated the rate by looking at the patient [11].

Though using ‘spot’ assessments appears to be common practice for some clinical staff, we could not identify any research evaluating the accuracy of this method. Given the importance of RR assessment, establishing the accuracy of the methods being used by doctors is extremely important.

Here we assessed the accuracy of hospital doctors in using both ‘spot’ and formal assessment of respiratory rate using videos depicting different respiratory rates. In this paper the term accuracy refers to the range, systematic error (bias), and imprecision of assessments.

## Aim

To investigate the accuracy of ‘spot’ and ‘formal’ assessments of RR by doctors in a central London teaching hospital, using videos depicting a mock patient with a known, constant RR.

## Methods

Doctors from the Royal Free Hospital in London invited to participate in the study at the end of meetings, including the care of the elderly and liver transplant departmental meetings and junior doctor teaching sessions. The purpose of the study was explained and participation completely optional.

Participants were given a questionnaire related to the importance of RR as a clinical sign, how they usually assess it, and whether they think it recordings are accurate. Participants were requested not to discuss or copy the answers of others. Next, participants watched three videos of a seated mock patient and asked to do a ‘spot’ assessment. Each video showed the individual breathing at a different, but constant rate and regular rhythm, with respiratory rates of respectively 30 (video A), 6 (video B) and 72 breaths/min (video C). The ‘true’ value stated was ensured using a muted metronome with visual display, to which the mock patient in the videos coordinated their respiratory rate. This also ensured a regular rhythm and rate throughout. Only 3 videos were shown to reduce bias from practice through multiple sequential assessments. No videos depicting ‘normal’ (i.e. 12–20) respiratory rates were used because, as stated above, we limited the study to three videos and decided investigating the ability to identify abnormal was more important. Furthermore, we wanted to investigate the accuracy of individual assessments of RR—we were not assessing the doctors’ ability to differentiate normal verse abnormal *between the videos*. For ‘spot’ assessments participants had 12 s to make their assessment. However, they were not told the specific length of the clip and were not able to use any form of timer for calculation. After having recorded estimations for each of the videos, their ‘spot’ assessments were collected to avoid any temptation to retrospectively alter answers.

Participants were then shown the videos again and given time to formally assess RR with a visible second counter. For ‘formal’ assessment we suggested assessment methods recommended in the hospital guidelines on RR assessment which advised counting for 30 s and multiplying by two (as long as the rate and rhythm were regular and constant); or training manuals and advisory articles which were to count for a full minute [9, 12]. We did not specify which method participants should use, but requested they use the method they normally use in their clinical practice. This time, videos were shown for 70 s, and assessments collected. Questionnaires were completed independently and anonymously.

The median and interquartile range (IQR) estimation for the two methods of assessment were calculated, as well as the bias and imprecision. “Bias”, also called “Systematic error” refers to the mean difference between the measured and known value. “Imprecision”, also called “Random error” is the standard deviation of the difference between measured values and the known value. A further interest was to evaluate the proportion of clinicians whose assessments correctly identified the RR shown in the videos as abnormal (i.e. outside of the normal range) for both the spot and the formal assessment. We used the range of 12–20 breaths/min as the normal range in keeping with recent UK Royal College of Physicians guidelines (2012) [13]. The McNemar test was used to evaluate whether the proportion of people who correctly identified RR shown in the videos as abnormal, both individually and overall, was significantly different using the ‘spot’ and the formal assessment. Chi square was used to assess for an association between correctly identifying the RR as abnormal and years of experience (if the expected values of one-fifth of the cells was <5 the Fisher exact test was used). One sided Cochran–Armitage trend test was used to evaluate whether there was a decreasing trend in the ability of identifying the RR as abnormal as the years of experience increase (if the expected values of one-fifth of the cells was <5 it was not reported because it is not valid in this circumstances). The variable years of experience was stratified in three categories: <1 year of experience, 2–10 years and >10 years.

## Results

Data were collected between May and July 2013. Fifty-four doctors participated, one declined to take part. 36 % (n = 18) had <1 year of experience, 40 % (n = 20) had 2–10 years and the remaining 24 % (n = 12) >10 years (See Table 2). The vast majority (93 %, n = 50) responded that RR is a ‘very important’ marker of a sick patient, with the remaining 7 % stating ‘fairly important’. 52 % stated they use spot assessments.

None of the participants thought that RR in medical notes are accurate all of the time, with 20 % (n = 11) stating most of the time; 72 % (n = 39) ‘sometimes accurate’; and 7 % (n = 4) ‘never accurate’.

**Fig. 1:**
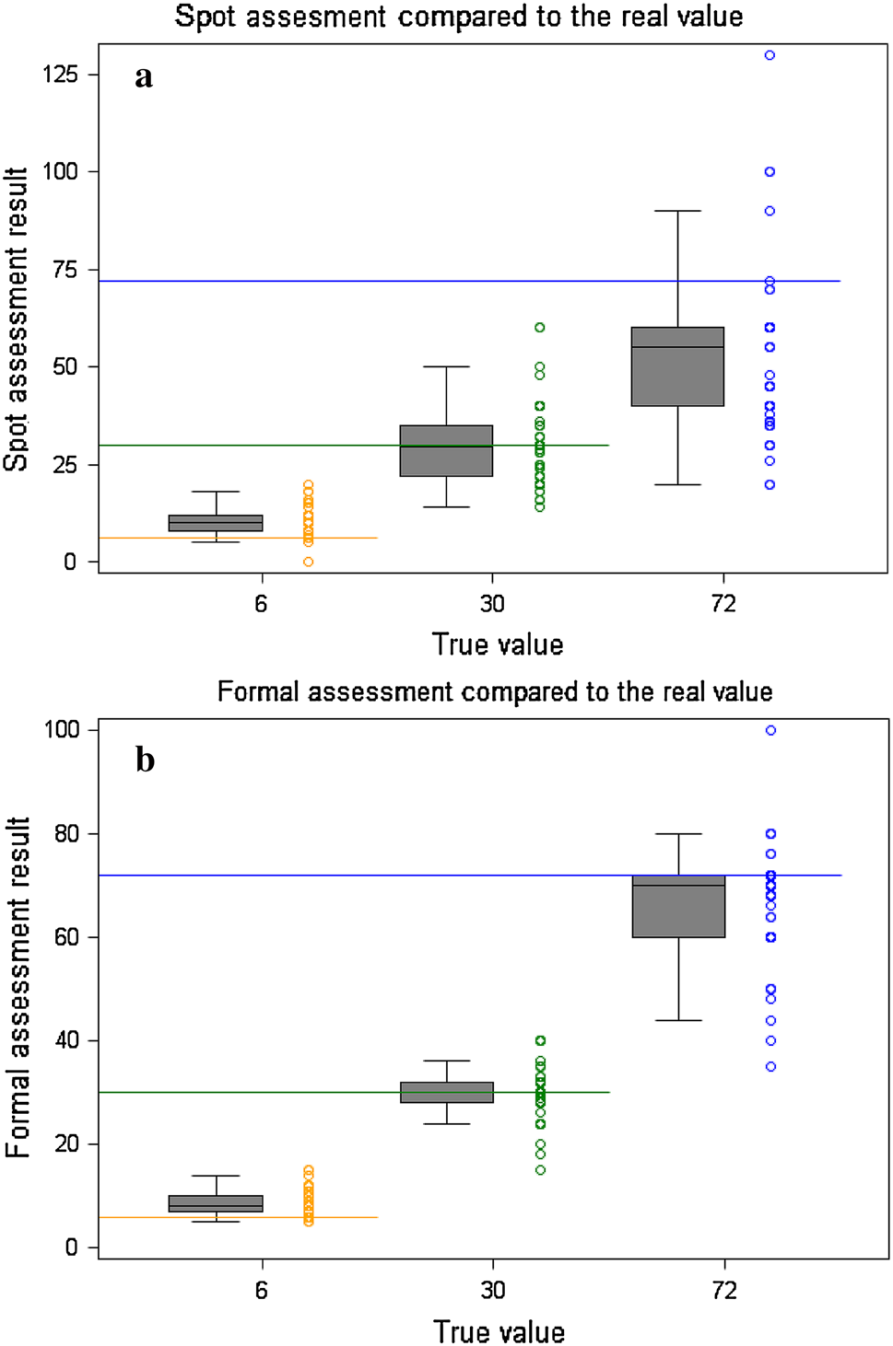
Box and whisker plots showing the median and interquartile ranges for ‘spot’ and formal assessments

Figure 1a shows the distribution of results from the spot assessment in relation to each video. The true value for each video is shown on the horizontal line, with the box and whisker plots showing the median and interquartile ranges. Figure 1b shows the equivalent results for the formal assessment.

Table 1 shows bias and imprecision of spot and formal assessments. The bias for the video showing 6 breaths/min is 4.4, this means that on average people when using spot assessment provide a value of 4.4 breaths more per minute than the real value, while the formal assessment provides a value of 2.46 breaths more per minute than the known value. This means that clinicians on average would estimate a value of 10.42 and 8.46, rather than true value of 6. When looking separately at the other videos the bias is very small for the video with a RR of 30 breaths/min: −0.28 and −0.02 for respectively spot and formal assessment. While for high RR, such as 72 breaths/min in this exercise, clinicians tend to underestimate the RR on average 19 breaths/min less than the real value using spot assessment, and 5 breaths/min less using formal assessment.

**Table 1:** Bias and imprecision of spot and formal assessment

Video	Known value	Spot assessment			Formal assessment		
		Mean difference between measured and known value	SD of the difference between measured values and the known value	Inaccuracy interval	Mean difference between measured and known value	SD of the difference between measured values and the known value	Inaccuracy interval
A	30	−0.28	10.31	(−24.8, 24.3)	−0.02	4.89	(−11.7, 11.6)
B	6	4.42	3.51	(−3.9, 12.8)	2.46	2.39	(−3.2, 8.1)
C	72	−19.18	20.20	(−67.2, 28.9)	−5.43	10.71	(−30.9, 20.0)

*SD* standard deviation

The imprecision of the ‘spot’ and formal assessments increases as the RR increases, and is consistently higher using the ‘spot’ compared with formal assessment.

**Fig. 2:**
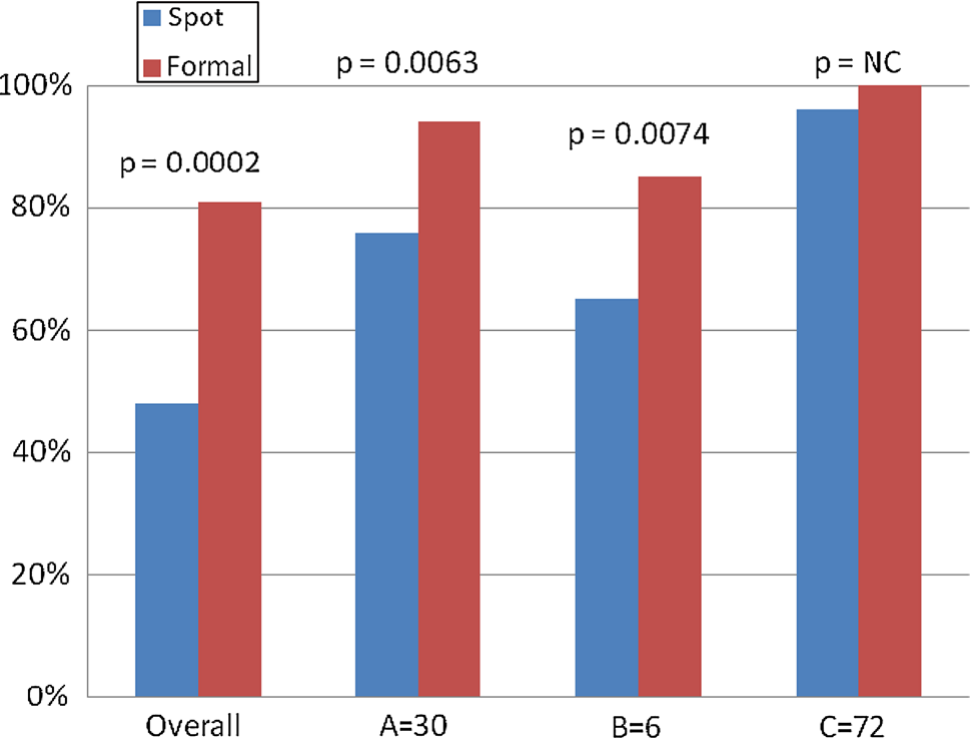
The percentage of clinicians who correctly identified the videos as showing a RR outside of the normal range (12–20 breaths/min) using ‘spot’ and formal assessments

Figure 2 shows the percentage of clinicians who correctly identified the videos as showing a RR outside of the normal range (12–20 breaths/min) using ‘spot’ and formal assessments. The column labelled ‘overall’ refers to the percentage of clinicians who correctly identified that all three of the videos showed abnormal RR. Using ‘spot’ assessment only 48 % of clinicians correctly identified all the three RR shown in the videos as abnormal, though this increases significantly to 81 % when using the formal assessment, nevertheless even using a formal assessment 19 % were not able to detect all three videos as abnormal. 100 % of the clinician identified the video showing a RR of 72 breaths/min as abnormal using the formal assessment and 96 % using the spot assessment. The RR which the least clinicians identified as abnormal was 6 breaths/min, which only 65 % using ‘spot’ assessment and 85 % using formal assessment correctly reported as being below 12 breaths/min.

Table 2 shows that number of years of experience has no effect on the accuracy of formal assessments (*p* = 0.6382). It also shows that doctors with more years of experience are less accurate at ‘spot’ assessments (*p* = 0.0490).

**Table 2:** Relationship between years of experience and accuracy of assessment

Years of experience	N	%	Number of people (%) who correctly identified as abnormal (Normal 12–20)	
			Spot assessment	Formal assessment
1	18	36	11 (61 %)	16 (89 %)
2–10	20	40	11 (55 %)	17 (85 %)
>10	12	24	3 (25 %)	9 (75 %)
*p* value	–	–	0.1295*, 0.0490***	0.6382**

* Chi square test

** Fisher exact test

*** one sided Cochran–Armitage trend test

After participating in the study, and having feedback regarding the actual RR represented in the videos, 59 % (n = 32) stated they were more likely to measure RR formally. 33 % (n = 18) stated they would not change their future practice.

## Discussion

The participants perceive RR as very important, though think it is assessed and recorded inaccurately. Low levels of confidence in documented recordings of RR means clinicians are unable to confidently use this parameter to assess for changes in patients’ condition from when they were last assessed, which has important implications for patient care.

The use of ‘spot’ assessments was reported by approximately half of participants, suggesting this practice is widespread. Both ‘spot’ and formal assessments were inaccurate, but formal assessments were consistently more so. This was reflected in the bias for ‘spot’ assessment ranging from 1.8 to 14 times that of the formal assessment.

The low proportion of participants able to identify all the three videos’ RR as abnormal (48 %) is an important finding. Prior to the study, many participants felt that the ‘spot’ assessment was sufficient to identify brady- or tachypnoeic patients, however these results suggest the contrary. Even using a formal assessment, many participants (18 %) were unable to identify that the rates shown were abnormal. As no assessments of ‘normal’ respiratory rates were made, conclusions cannot be drawn regarding sensitivity and specificity for detecting abnormality.

The decreasing accuracy of ‘spot’ assessments with increasing clinical experience may reflect differing roles of senior and junior doctors. Junior doctors maybe assessing RR more frequently than consultants in their current roles. Alternatively it may be that junior doctors use ‘spot’ assessments more than their senior colleagues and are subsequently more practiced.

A potential criticism of this study is that we did not specify which method should be used for the formal assessment therefore multiple different methods could have been used. Participants were advised to use the method that they usually used on the wards, in order to gain results representative of their practice.

A further limitation is that in reality RR is not measured in isolation, rather it is assessed in the context of the entire patient who maybe showing other signs of respiratory distress. These additional factors would be considered with RR to gain an overall impression of the patient. Therefore a video of an otherwise ‘well’ patient with an isolated abnormal RR could be seen as unrealistic. In response to this point, RR is often the first clinical parameter to become abnormal and indicate a change in a patient’s condition. Furthermore, particularly in younger patients, physiological compensation can be such that other clinical signs remain within normal parameters until late, creating a situation where signs indicative of deterioration may be in isolation. In addition, if participants are unable to measure RR accurately when it is shown in isolation, it is unlikely that they would be able to accurately assess it in a more holistic assessment.

Not including normal a RR in the data collection limits the extent to which quantification of diagnostic accuracy is possible, specifically, regarding identifying normal Vs abnormal. This does not invalidate the key findings, but highlights a future avenue of research.

The effect of inaccurate measurement of RR on patient outcomes remains unclear. It can be logically extrapolated that as respiratory rate is considered an important physiological parameter in the clinical assessment of patients, inaccurate assessments would negative impact upon patient outcomes. However, the extent of this impact has not been investigated here.

A further limitation is that this study looked only at doctors, while in practice, the majority of RR recordings in observation charts are done by nursing staff. Doctors were targeted here as previous research found doctors, and not nursing staff, stated that they used ‘spot’ assessments which we wanted to investigate. Further research comparing the accuracy of different staff groups could be useful, as it would help direct educational interventions where most needed.

## Conclusions

This research shows that methods of RR assessment currently used by doctors are inaccurate. We have shown that ‘spot’ assessment is highly inaccurate, to the extent that the majority of doctors were unable to reliably identify abnormal RR. This study provides evidence against the proposition by some doctors that ‘spot’ assessments accurately identify tachypnoea or bradypnoea. The inaccuracy of ‘spot’ assessments appears to increase with years of clinical experience but the explanation for this finding remains unclear. Formal methods of assessment appear to be more accurate than spot assessments. However, 18 % of people were still unable to reliably identify abnormal respiratory rates in all three videos presented using formal assessment. The inaccuracy of assessment is likely to have negative implications for patient care, and subsequently patient outcomes.

RR is a key component of assessing a patient in multiple contexts, with NICE stating it is the most sensitive marker of a deteriorating patient, and often the first sign of deterioration in a patient’s condition [1]. Therefore valuable clinical information is not being used that could prompt both rapid identification and response to clinical need. Arguably, beyond simply delaying care, inaccurate recordings may provide false assurance that a respiratory rate is normal, when in fact it is not, and therefore actively delay care and lead to inappropriate clinical decisions.

Further research assessing the accuracy of specific methods of RR assessment would be useful, as would research on clinical outcomes. This research highlights an important aspect of clinical care which is currently being performed poorly. Immediate recommendations include the exclusive use of formal assessment of RR, with longer term improvements through educational initiatives, are warranted.

## Electronic supplementary material

Below is the link to the electronic supplementary material.

Supplementary material 1 (MOV 1743 kb)

Supplementary material 2 (MOV 2323 kb)

Supplementary material 3 (MOV 1829 kb)

## References

[1] NICE CG50: Recognition of and response to acute illness in adults in hospital. National Institute of Clinical Excellence. http://www.nice.org.uk/CG50 (2007). Accessed 02 Feb 2014. 21204323

[2] Fieselmann JF, Hendryx MS, Helms CM (1993). Respiratory rate predicts cardiopulmonary arrest for internal medicine inpatients. J Gen Intern Med.

[3] Hodgetts TJ, Kenward G, Vlachonikolis IG (2002). The identification of risk factors for cardiac arrest and formulation of activation criteria to alert a medical emergency team. Resuscitation.

[4] Goldhill DR, McNarry AF, Mandersloot G (2005). A physiologically-based early warning score for ward patients: the association between score and outcome. Anaesthesia.

[5] Subbe CP, Davies RG, Williams E (2003). Effect of introducing the Modified Early Warning score on clinical outcomes, cardio-pulmonary arrests and intensive care utilisation in acute medical admissions. Anaesthesia.

[6] Hogan H, Healey F, Neale G (2013). Preventable deaths due to problems in care in English acute hospitals: a retrospective case record review study. BMJ Qual Saf.

[7] Bone RC, Balk RA, Cerra FB (1992). Definitions for sepsis and organ failure and guidelines for the use of innovative therapies in sepsis. The ACCP/SCCM Consensus Conference Committee. American College of Chest Physicians/Society of Critical Care Medicine. Chest.

[8] BTS/SIGN: British Guideline on the management of asthma. British Thoracic Society. https://www.brit-thoracic.org.uk/guidelines-and-quality-standards/asthma-guideline/ (2012). Accessed 02 Feb 2014.

[9] Dougherty L, Lister S, Mallett J, Dougherty L (2008). Supporting the patient through the diagnostic process: Observations. The Royal Marsden NHS Trust manual of clinical procedures.

[10] Lovett PB, Buchwald JM, Sturmann K, Bijur P (2005). The vexatious vital: Neither clinical measurements by nurses nor an electronic monitor provides accurate measurements of respiratory rate in triage. Ann Emerg Med.

[11] Philip K, Richardson R, Cohen M (2013). Staff perceptions of respiratory rate measurement in a general hospital. Br J Nurs.

[12] Moore T. Respiratory assessment in adults. Nurs Stan. 2007;21: 48–56. http://www.snjourney.com/ClinicalInfo/Systems/Resp/RespAss.pdf. Accessed 02 Feb 14. 10.7748/ns2007.08.21.49.48.c4605 17844906

[13] Royal College of Physicians. National Early Warning Score (NEWS): Standardising the assessment of acute-illness severity in the NHS Report of a working party July 2012. http://www.rcplondon.ac.uk/sites/default/files/documents/national-early-warning-score-standardising-assessment-acute-illness-severity-nhs.pdf. Accessed 02 Feb 14.

